# Supplementary material for: Genomic perspective on the bacillus causing paratyphoid B fever
Source: Nat Commun. 2024 Dec 10;15:10143. doi: 10.1038/s41467-024-54418-4 (PMC11632088; doi:10.1038/s41467-024-54418-4)
Supplement: Supplementary file 1 — Supplementary Information [file 41467_2024_54418_MOESM1_ESM.pdf]

Supplementary Information

Genomic perspective on the bacillus causing paratyphoid B fever

Jane Hawkey, Lise Frézal, Alicia Tran Dien, Anna Zhukova, Derek Brown, Marie Anne Chattaway, Sandra Simon, Hidemasa Izumiya, Patricia I. Fields, Niall de Lappe, Lidia Kaftyreva, Xuebin Xu, Junko Isobe, Dominique Clermont, Elisabeth Njamkepo, Yukihiro Akeda, Sylvie Issenhuth-Jeanjean, Mariia Makarova, Yanan Wang, Martin Hunt, Brent M. Jenkins, Magali Ravel, Véronique Guibert, Estelle Serre, Zoya Matveeva, Laëtitia Fabre, Martin Cormican, Min Yue, Baoli Zhu, Masatomo Morita, Zamin Iqbal, Carolina Silva Nodari, Maria Pardos de la Gandara, François-Xavier Weill

Supplementary Methods

Short-read sequencing

At NHSGGC, genomic DNA was extracted with the QIAsymphony system (Qiagen, Hilden, Germany), the libraries were prepared with the Illumina DNA Prep Kit (Illumina) and sequencing was performed with MiSeq (Illumina). At UKHSA, genomic DNA was extracted with the QIAsymphony system (Qiagen), the libraries were prepared with the Nextera XT kit (Illumina) and sequencing was performed with HiSeq 2500 (Illumina). At NIID, genomic DNA was extracted with the QIAseq FX DNA Library Kit (Qiagen), the libraries were prepared with the QIAseq FX DNA Library Kit (Qiagen), and sequencing was performed with MiSeq (Illumina). At UCD, genomic DNA was extracted with the EZ1® DNA Tissue kit

(Qiagen), the libraries were prepared with the Nextera DNA Flex library prep kit (Illumina), and sequencing was performed with MiSeq (Illumina). At RKI, genomic DNA was extracted with the GenElute™ Bacterial Genomic DNA Kit (Sigma-Aldrich, St. Louis, MO, USA) or by combined thermal and mechanical disruption with acid-washed glass beads (Sigma-Aldrich) in a TissueLyser II bead mill (Qiagen), the libraries were prepared with the Nextera XT kit (Illumina), and sequencing was performed with MiSeq (Illumina) or NextSeq 500 (Illumina).

Genomic dataset for non-SPB$^-$ PG1 *Salmonella* genomes

For assessment of the new SNV-based genotyping tool on *Salmonella* genomes other than *d*-tartrate-nonfermenting *Salmonella enterica* serotype Paratyphi B of phylogroup 1 (SPB$^-$ PG1), we assembled a genomic dataset for 102 representative *Salmonella* reference genomes present in EnteroBase (**Supplementary Data 10** and https://enterobase.warwick.ac.uk/species/senterica/search_strains?query=workspace:120031) All but two were from non-redundant strains from the SARA and SARB reference collections of *S. enterica*[1]. The remaining two genomes were from *S. enterica* serotype Paratyphi A strains ATCC 9150 and ATCC 11511 (the genome of SARB42, the only strain of this serotype in the SARA and SARB collections was not found in EnteroBase). Thirty-five different *S. enterica* serotypes were represented in our genomic dataset, including 8 SPB$^+$ (from PG2, PG4 and PG9). The short-read assemblies downloaded from EnteroBase were used as input for our SNV-based genotyping tool.

Supplementary Notes

Epidemiology of paratyphoid B fever during the first half of the 20th century

From its discovery in France in 1896 until 1903, 69 cases of paratyphoid B fever (PTB) — mostly sporadic or small-scale outbreaks — were reported in various European countries and the United States of America (US)[2,3]. In the United Kingdom (UK), in 1906, it was estimated that 3% of the 3,000 typhoid fever cases notified each year in London could be PTB cases; in Germany in 1901 and 1905, this percentage was around 7%[4]. By contrast, in the US, no *Salmonella enterica* serotype Paratyphi B (SPB) was isolated from 250 cases of enteric fever (200 of typhoid fever and 50 of paratyphoid A) in Philadelphia in 1908-1909 (ref.[3]). Between December 1914 and February 1915, 6.7% of the ~4,500 enteric fever cases seen at the Military Hospital of Zuydcoote, France were PTB cases[5]. A new combined vaccine (TAB) — extending the initial vaccination against typhoid fever to paratyphoid A and B fevers — was therefore introduced in 1915-1916 for Allied forces[6]. At the end of the 1930s, PTB became much more prevalent than typhoid fever in England[7]. Between 1923 and 1941, the vast majority of the 40 outbreaks of PTB, involving a total of more than 4,200 cases (from 4 to 883 cases per outbreak) reported in the UK were due to food contaminated by transient or chronic carriers[8]. The foods contaminated were natural or synthetic cream (an emulsion of vegetable oils or fat with water, with or without the addition of other substances of dairy or non-dairy origin), unpasteurised milk, ice-cream, bakery products or confectionery[8]. One contaminated synthetic cream, in particular, was implicated in seven outbreaks between 1940 (when the sale of natural cream was forbidden) to 1941 and resulted in 1,462 cases and at least 10 deaths. After World War II, commercial bakeries in the UK were infected via a new source: contaminated frozen whole egg imported from China[9,10]. Contaminated water, either alone or on edible plants, was less frequently implicated than food items[8,11]. Interestingly,

whereas typhoid fever is restricted to humans, two reports from Scandinavia (Norway in 1937 and Sweden in 1938) mentioned dogs as sources of SPB$^-$ infections in humans[8].

Validation of the SPB$^-$ PG1 diversity dataset

Our serotype prediction approach identified one genome among the 568 genomes of the diversity dataset that did not correspond to SPB (the 116K strain, see below). Twenty-one genomes were identified as monophasic SPB (without the *fljB* gene encoding the H2 antigen "1,2"), and it was not possible to predict the O antigen for five genomes due to a low read coverage in the corresponding region (these five genomes had the correct *fliC* and *fljB* genes, encoding, the "b" and "1,2" antigens, respectively, and had been phenotypically serotyped as SPB at Institut Pasteur) (**Supplementary Data 1**). The rest of the genomes were inferred to be SPB. We then checked that these 568 genomes belonged to PG1, the invasive lineage of SPB, described by Connor and coworkers[12]. We used the EnteroBase core-genome MLST (cgMLST) scheme — based on 3,002 core genes — which had been successfully used to study the population structure of *Salmonella enterica*[13,14] to confirm that our 568 genomes belonged to invasive PG1, by establishing a link between the cgMLST and PG data. After curation of the genomes and metadata described by Connor and coworkers[12] (see Methods section "Genomic typing methods") (**Supplementary Data 7**), a tree based on the cgMLST allelic distance for these genomes made it possible to recognise the 10 known PGs of SPB (**Supplementary Fig. 1a**). Following the hierarchical clustering[15] of cgMLST data, also implemented in EnteroBase, all isolates assigned to PG1-PG5 clustered in HC2000_155 and HC900_155. However, the PG1 isolates could be distinguished from PG2 to PG5 isolates at the HC400 level (i.e., grouping together genomes with no more than 400 allelic differences). All the invasive PG1 genomes, and only these genomes, belonged to HC400_1620 (**Supplementary Fig. 1b**). Furthermore, only the HC400_1620 genomes contained the

specific SNV within STM 3356 described in SPB$^-$ strains[16]. All 446 genomes from SPB$^-$ isolates and strains contributed by various reference laboratories across the world for this study and the 109 previously published genomes belonged to HC400_1620 and contained the *d*-Tar$^-$ specific SNV (**Supplementary Data 1**).

We also used a combination of this HC400_1620 level and the presence of the *d*-Tar$^-$ specific SNV to search for additional unpublished SPB$^-$ genomes in EnteroBase, a very large genomic database containing >400,000 *Salmonella* genomes at the time of study. This search captured 12 additional genomes from reference strains (e.g., SARA collection)[17] or from isolates collected locally in regions of the world not well covered by our initial dataset (**Supplementary Data 1**). During this search, we unexpectedly found within HC400_1620 a reference strain (116K) of an extremely rare serotype, Onarimon (antigenic formula: 1,9,12:b:1,2), deposited independently by one of the participating laboratories (Institut Pasteur). Serotype prediction based on genomic sequence confirmed this serotype and the *d*-Tar$^-$ specific SNV was present. There were only 28 serotype Onarimon strains reported in 1965 (among the 547,386 strains from diverse sources across the world)[18] and this serotype has been reported to cause paratyphoid fevers[19]. As in *Salmonella* spp., serotype antigens can be subject to horizontal gene transfer and homologous recombination[20]; we therefore considered 116K to be an O antigen-variant of SPB$^-$ and we included it in the study.

For one unpublished genome (ATCC 10719, original name 41-H-6) we were unable to confirm membership of HC400_1620 because it was a draft genome prepared from 454 sequences and was not, therefore, accepted by EnteroBase (**Supplementary Data 1**). However, this genome came from an old SPB strain used to prepare the US Army TAB

vaccine in 1940 (ref.[21]) and it contained the *d*-Tar$^-$ specific SNV. We therefore retained this genome in the study.

Genotypes found in non-human SPB$^-$ PG1 isolates

Twelve different genotypes were observed for the 14 animal isolates (from six molluscs, two insects, two dogs, one squirrel, one pig, one bird and one crustacean) and 14 genotypes were observed for the 42 environmental isolates (mostly from river water). These genotypes were identical to those found in human isolates collected in the same geographic region. The first dog isolate (#1190) was obtained from a dog reported sick (diarrhoea and spontaneous abortion) in the week before the onset of three human cases in a Swedish village in 1938 (refs.[22,23]). This isolate belonged to genotype 4. The second dog isolate was collected in Algeria (North Africa) in 1966 and belonged to genotype 7.3.1_NorthAfrica1. The only two food isolates studied were isolated in Iraq in 1976 from locally prepared food[24] and were of genotype 9.0, which was mostly isolated in Western Europe. The other four Iraqi isolates, obtained from humans between 1974 and 1980, belonged to another three genotypes (10.3.1_SouthAsia1, 10.3.2_MiddleEast1, and 10.3.8.4_MiddleEast4).

Prophages of SPB$^-$ PG1

We facilitated the pan-genome analysis, including the assignment of accessory genes to clearly delineated prophages (**Supplementary Data 8**), by also including in our analysis the complete genomes of 14 isolates from the "diversity dataset", which consisted of 12 genomes generated for this study (including the reference genome, CIP 54.115) and two publicly available genomes (P7704 and SARA41_FB_1) (**Supplementary Data 1**).

At least three sorts of non-*sopE* prophages could occupy insertion site "A". This site is located right next to the *hin-fljB-fljA* region, which is involved in the expression of the phase

2 flagellin (“1,2” antigen encoded by *fljB*) (**Supplementary Fig. 13**). Prophage rearrangement at site “A” leading to the loss of the of *hin-fljB-fljA* region might account for the appearance of monophasic isolates (i.e., lacking phase 2, with the antigenic formula 4:b:-) in genotypes 4 and 7.3 (**Supplementary Data 1**).

Development of a new SNV-based genotyping tool for SPB^{-} PG1

Our genotyping scheme for SPB^{-} PG1 implemented in Mykrobe software yields the result “Salmonella_enterica” or “unknown” under the column “phylo-group” of the Mykrobe output table if the *Salmonella*-specific *invA* gene is detected (for *Salmonella* genomes) or not detected (for non-*Salmonella* genomes), respectively. The genotyping tool yields the result “Salmonella_ParatyphiB” under the column “species” of the Mykrobe output table if the *d*-Tar^{-} SNV marker is present (for SPB^{-} PG1 genomes). The absence of a call for the *d*-Tar^{-} SNV marker — indicated as “unknown” under the column “species” — may result from the presence of the *d*-Tar^{+} SNV (for non-SPB^{-} PG1 genomes, in particular SPB^{+} genomes), a low read coverage for the *d*-Tar^{-} SNV marker region (for SPB^{-} PG1 genomes), or even, theoretically, a deletion of the *d*-Tar SNV marker region in rare strains of *Salmonella* spp.. The absence of a call for the *d*-Tar^{-} SNV marker does not prevent a final genotype call being obtained (result under the column “lineage” of the Mykrobe output table).

For validation of our scheme, we first analysed the 568 genomes of our diversity dataset. A concordance of 100% was obtained between the genotypes assigned by Mykrobe and those initially defined on the basis of both hierBAPS and visual inspection (**Fig.2a,b**). The scheme was then used on the surveillance dataset containing 336 routinely obtained genomes (111 already present in the diversity dataset and 225 new genomes) from public health laboratories in four countries (UK, *n* = 200; France, *n* = 84; USA, *n* = 39; Canada, *n* = 13), with isolation

dates between 2014 and 2023 (see Methods section "*S. enterica* serotype Paratyphi B sequence data collection") (**Supplementary Data 6)**. The SNV-based genotyping scheme accurately captured the population structure as defined by a core-genome phylogeny on the 793 genomes from both datasets (**Supplementary Fig. 11).** Three new routinely sequenced genomes, without travel information, from the US and Canada were assigned to genotype 10.3.8. However, on the basis of phylogeny, one (PNCS011535) of these genomes was considered to be intermediate between genomes typed as 10.3.8 and those typed as 10.3.8.4_MiddleEast4, and the other two (PNUSAS023302 and PNUSAS023173) were grouped together and considered intermediate between genomes typed as 10.3.8 and those typed as 10.3.8.1_SouthAsia2. If similar isolates were to be identified in the future, we would perhaps have to refine the definition of genotype 10.3.8 slightly. Only one of the 793 genomes genotyped (44-66) was not called by Mykrobe due to a missing *invA* gene. Fourteen other genomes were called correctly by Mykrobe despite read coverage being too low for the *d*-Tar^{-} SNV region, precluding formal identification of the *d*-Tar^{-} SNV marker (result reported as "unknown" in the "species" column).

The testing of this genotyping tool on a set of 102 representative genomes of *S. enterica* not belonging to SPB PG1 ("**Supplementary Methods "Genomic dataset for non-SPB^{-} PG1 *Salmonella* genomes"**) confirmed the presence of the *invA* gene and the absence of the *d*-Tar^{-} SNV in all 102 isolates (**Supplementary Data 10**). However, a genotype was called for 97 (95.1%) genomes: 92 genomes belonging to genotype 7.0 (indicated as "7" in the Mykrobe output table) and five belonging to genotype 7.3. Two genomes were genotyped as a mixture of genotypes 7.0 and 8.0, and three genomes (SPB+) were typed as "unknown". Therefore, to prevent a non-PG1 *Salmonella* genome being erroneously assigned to a PG1 genotype, we

strongly recommend ensuring that all *Salmonella* genomes genotyped with this scheme belong to cgMLST HC400_1620, a robust signature of SPB^{-} PG1.

Supplementary Table 1. Comparison of the *sopE* prophages found in the 14 complete SPB$^-$ PG1 genomes

Isolate	Genotype	Prophage insertion site	Prophage length	Prophage genus	Blastn query *	Query name	Query coverage (%)	Nucleotide identity (%)	#hit_genes from PHASTER
63-90	1	1 [SopE]	43665	*Brunovirus*	NC_028699.1	*Salmonella* phage SEN34	53	90.9	36
B76	2.1	1 [SopE]	43084	*Brunovirus*	NC_028699.1	*Salmonella* phage SEN34	45	89.8	30
CIP 54.100	2.1	1 [SopE]	43084	*Brunovirus*	NC_028699.1	*Salmonella* phage SEN34	45	89.8	30
CIP A214	4	1 [SopE]	43096	*Brunovirus*	NC_028699.1	*Salmonella* phage SEN34	45	89.8	30
B2590	9.1_France	1 [SopE]	43987	*Brunovirus*	NC_028699.1	*Salmonella* phage SEN34	44	95.9	33
B2590	9.1_France	2 [SopE]	41250	*Brunovirus*	NC_028699.1	*Salmonella* phage SEN34	50	93.7	29
SARA41	7.3.1_NorthAfrica1	2 [SopE]	43291	*Brunovirus*	NC_028699.1	*Salmonella* phage SEN34	55	91.9	37
B2227	7.3.2_BAOR	2 [SopE]	44615	*Brunovirus*	NC_028699.1	*Salmonella* phage SEN34	55	91.9	39
B624	6	2 [SopE]	41336	*Brunovirus*	NC_028699.1	*Salmonella* phage SEN34	36	95.9	26
P7704	10.3.6_SouthAmerica	2 [SopE]	43943	*Brunovirus*	NC_028699.1	*Salmonella* phage SEN34	38	95.9	31
B1655	10.3	2 [SopE]	43943	*Brunovirus*	NC_028699.1	*Salmonella* phage SEN34	38	95.9	31
B1727	7.3	2 [SopE]	43943	*Brunovirus*	NC_028699.1	*Salmonella* phage SEN34	38	95.9	31
B97	7.2_EuropeEasternAsia	2 [SopE]	43943	*Brunovirus*	NC_028699.1	*Salmonella* phage SEN34	38	95.9	31
B62	7.2_EuropeEasternAsia	2 [SopE]	43941	*Brunovirus*	NC_028699.1	*Salmonella* phage SEN34	38	95.9	31
CIP 54.115	7.3	2 [SopE]	43941	*Brunovirus*	NC_028699.1	*Salmonella* phage SEN34	38	95.9	30
B2227	7.3.2_BAOR	3 [SopE]	34723	*Xuanwuvirus*	NC_026014.1	Enterobacteria phage P88	83	97.9	44
B2590	9.1_France	3 [SopE]	34723	*Xuanwuvirus*	NC_026014.1	Enterobacteria phage P88	83	97.8	44
B624	6	3 [SopE]	34724	*Xuanwuvirus*	NC_026014.1	Enterobacteria phage P88	83	97.8	44

*best hit identified by PHASTER

Supplementary Table 2. GenBank accession numbers and co-ordinates of the genes studied to confirm that our isolates were SPB$^-$

Target	Strain	Accession no.	Coordinates
rfb_O4	*S. enterica* serotype Typhimurium str. LT2	NC_000913.3	2160595-2182675
rfb_O9	*S. enterica* serotype Enteritidis str. P125109	AM933172.1	2162790-2184501
fliC_b	*S. enterica* serotype Paratyphi B str. B62	CP147902	1931292-1932779
fljB_1,2	*S. enterica* serotype Paratyphi B str. B62	CP147902	1120688-1122208
***d*-Tar$^+$**	*S. enterica* serotype Paratyphi B str. NCTC 5706	AY211490.1	1-291*
***d*-Tar$^-$**	*S. enterica* serotype Paratyphi B str. NCTC 3176	AY211491.1	1-291*

*single-nucleotide variant (SNV) at position 252 (gene STM 3356): G (*d*-Tar$^+$) or A (*d*-Tar$^-$)

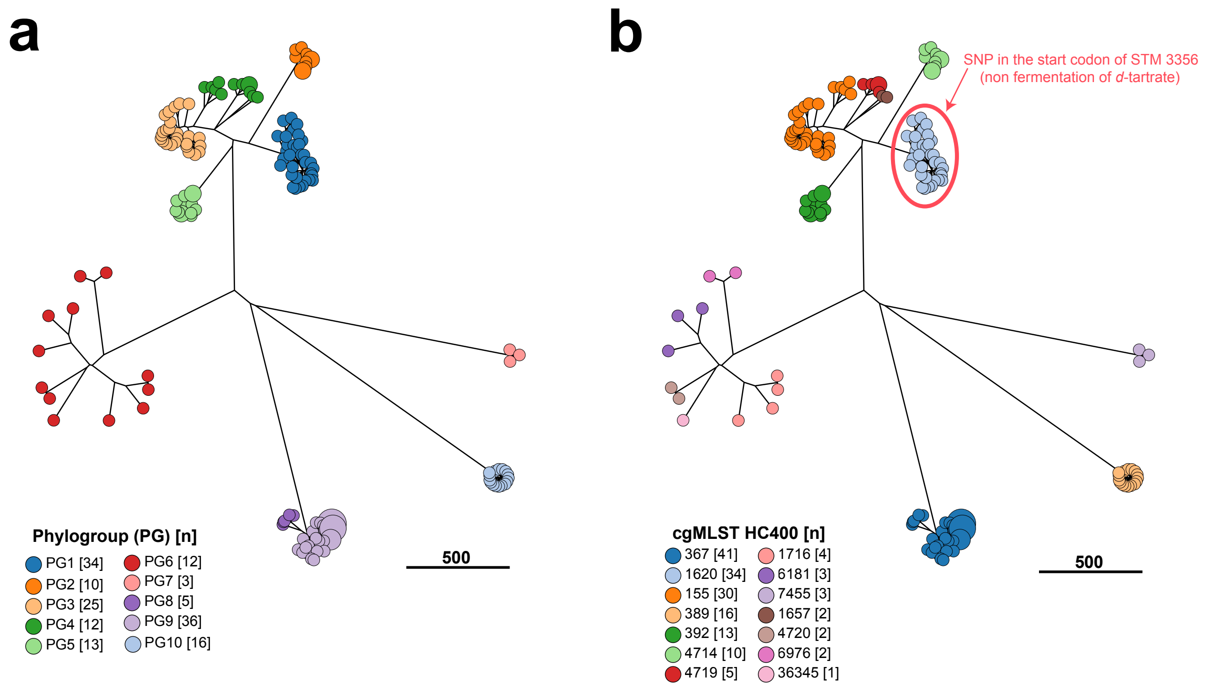

Supplementary Figure 1. A NINJA neighbour-joining GrapeTree of 166 SPB genomes described by Connor *et al.*[11]. **a,** The tree nodes are colour-coded by phylogroup (PG) (see the legend, inset). **b,** The tree nodes are colour-coded by cgMLST HC400 data (see the legend, inset). The presence of the specific SNV found in the STM 3356 gene of SPB$^-$ (ref.[15]) is indicated. The scale bars indicate the number of cgMLST allelic differences. The interactive version of the tree is publicly available from https://enterobase.warwick.ac.uk/ms_tree?tree_id=92077

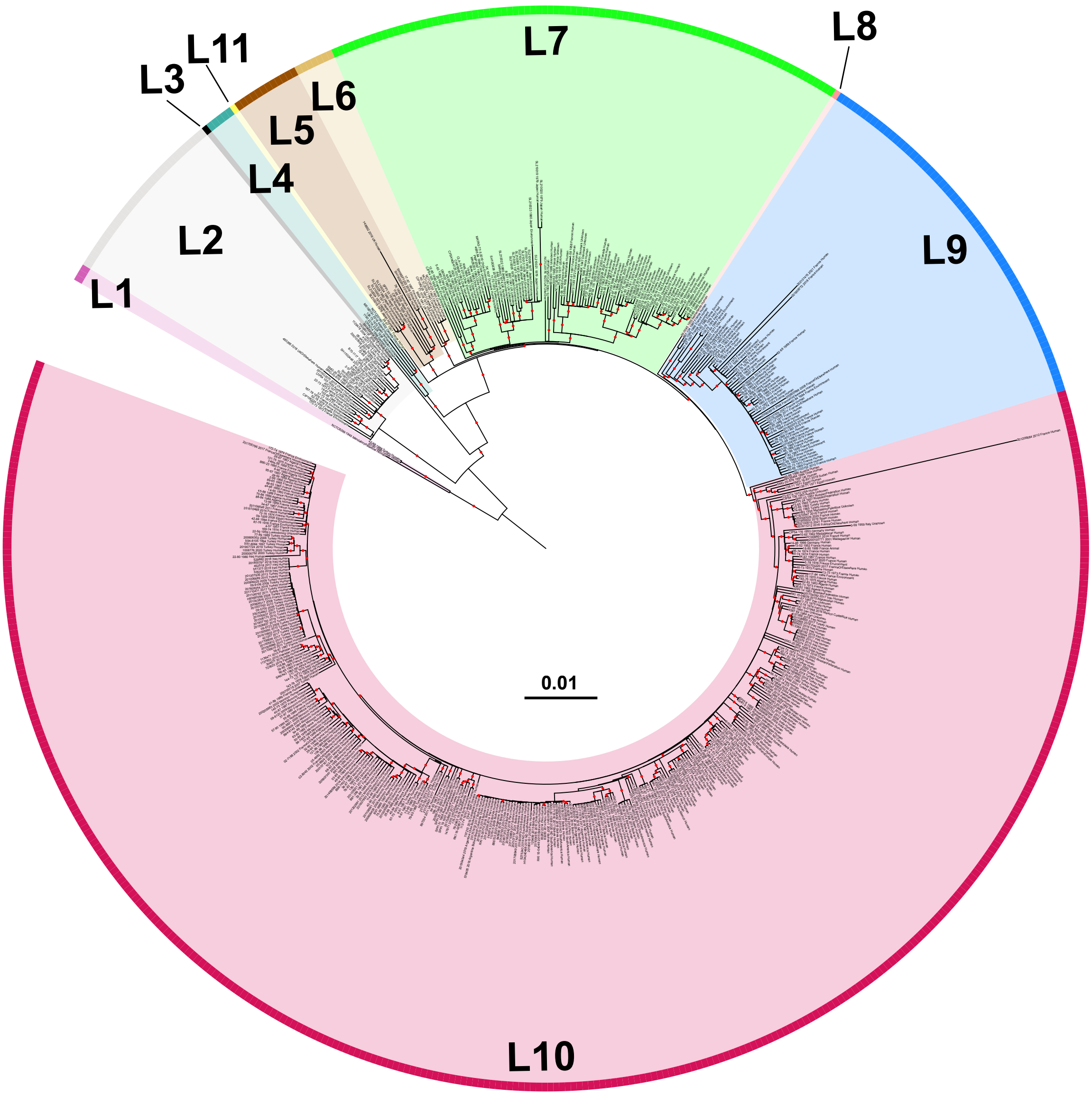

Supplementary Figure 2. Circular maximum likelihood phylogeny of the 568 SPB$^-$ PG1 genomes of the diversity dataset. Same phylogeny as in Fig. 1a, except that for each isolate, its name, year of collection, country of origin, and source, are shown at the tips of the tree when magnified. The lineages are also shown. Red dots indicate bootstrap values ≥95%.

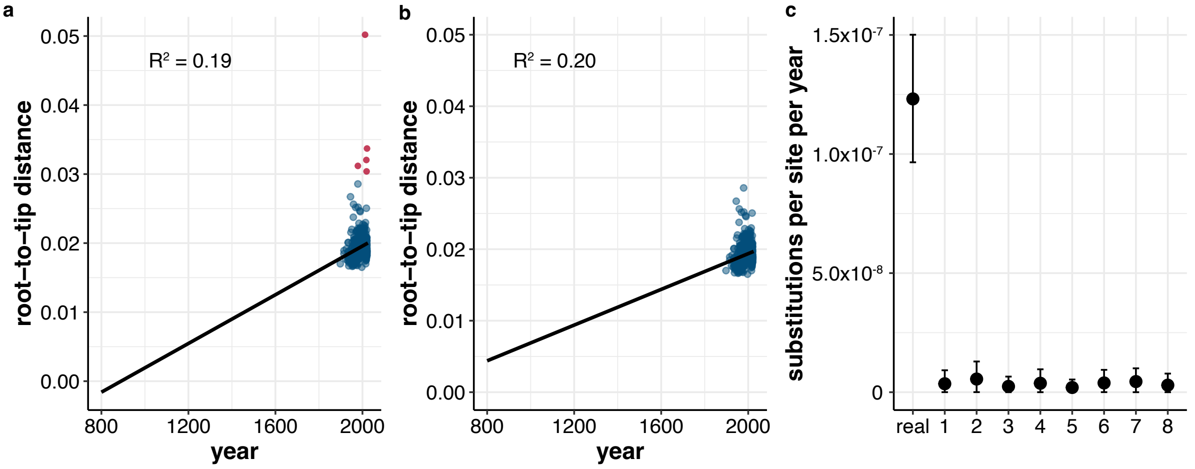

Supplementary Figure 3. Temporal structure of SPB^{-} PG1 genomes. a, Correlation of root-to-tip distances with year for all 568 genomes of the diversity dataset in the maximum likelihood phylogeny. Red points indicate outlier genomes, which were long branches with a distance > 0.03, and blue points represent all other genomes. R2 shows the Pearson correlation coefficient. **b,** Correlation of root-to-tip distances with year after excluding the outlier genomes shown in panel "a". **c,** Date randomisation test for the dated BEAST2 phylogeny. The first point indicates the median rate (in substitutions site^{-1} year^{-1}) estimated by BEAST2 for the real dates, with bars showing the 95% height posterior density (HPD) interval. Subsequent points show the rate estimates from eight independent BEAST2 runs in which dates were randomised across the phylogeny.

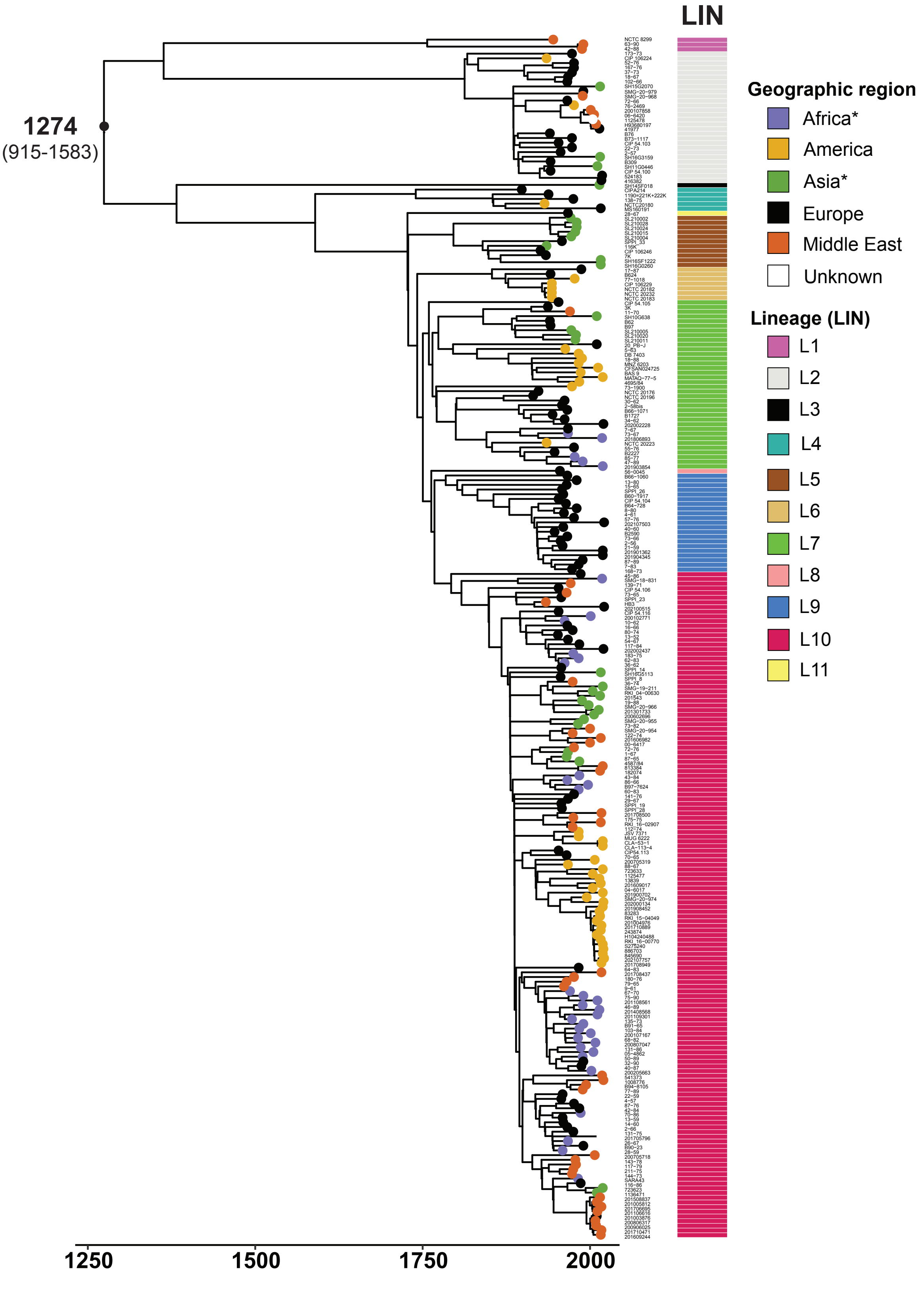

Supplementary Figure 4. Timed phylogeny of a representative subsample of 256 SPB^- PG1 genomes. Same phylogeny as in Fig. 2a, except that the name of the isolates is indicated.

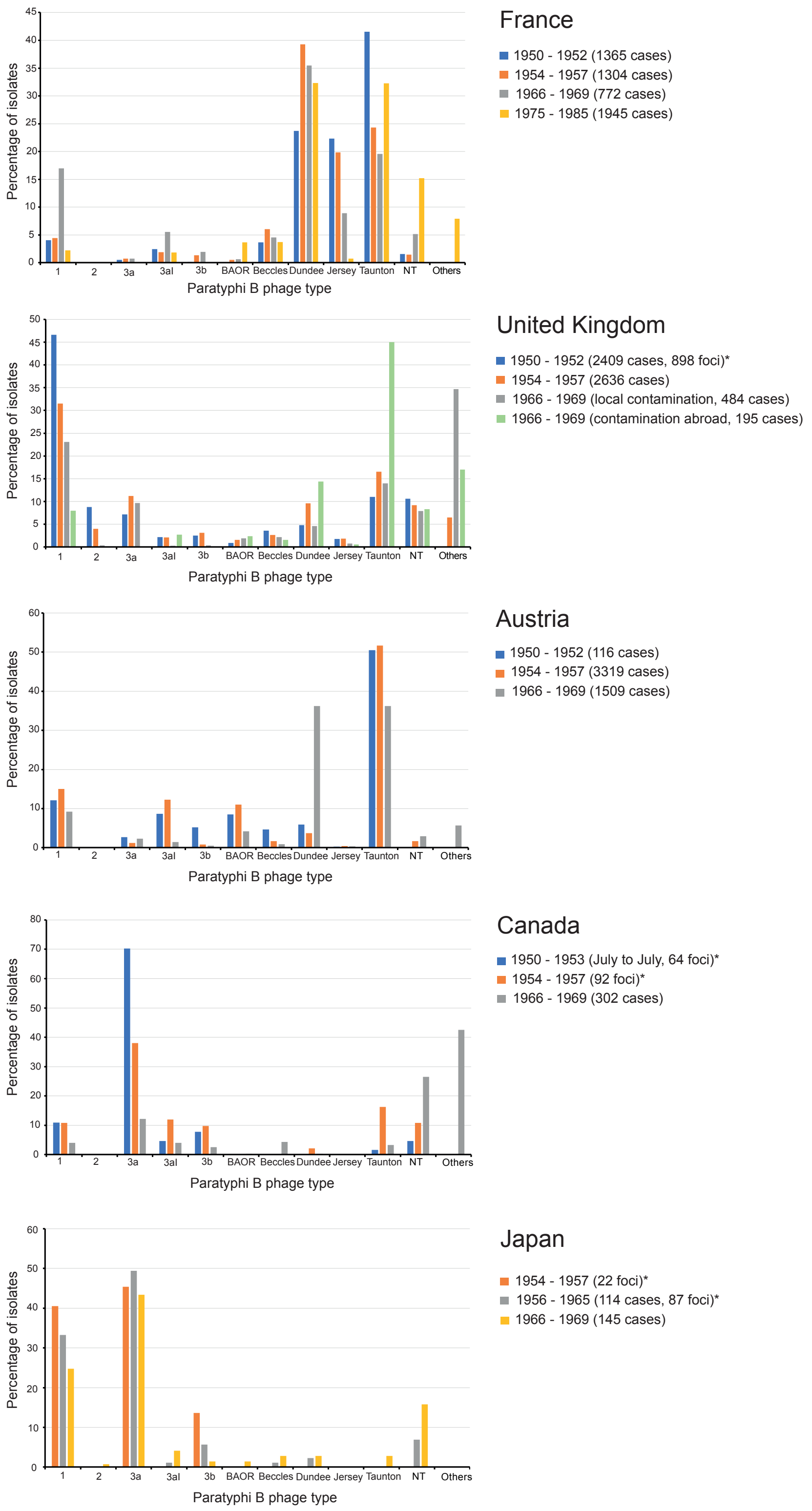

Supplementary Figure 5. Selected phage-typing data reported for SPB isolates from France, UK, Austria, Canada, and Japan. The original data can be found in refs[25-29].

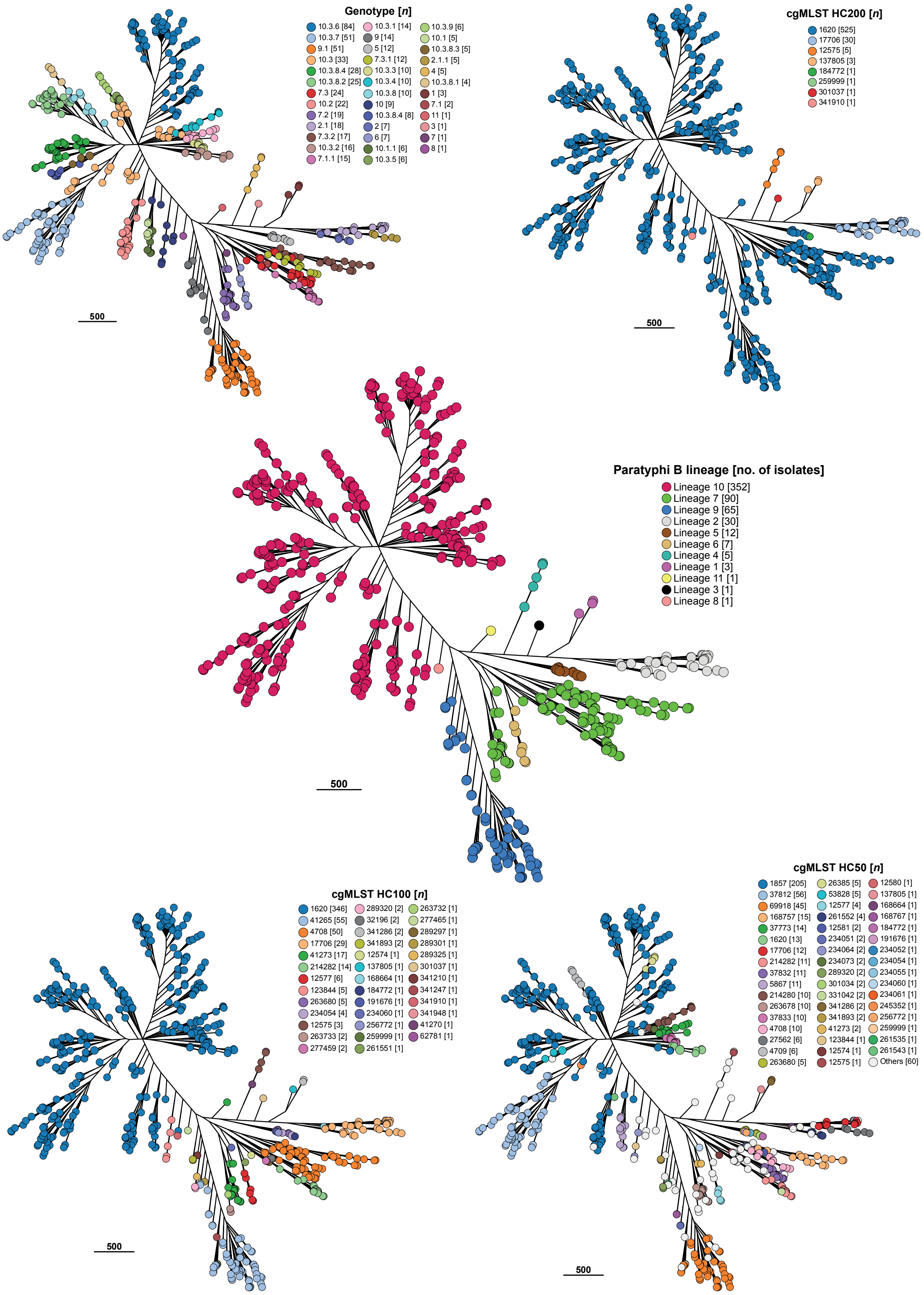

Supplementary Figure 6. A NINJA neighbour-joining GrapeTree for 567 SPB⁻ PG1 isolates from the diversity dataset. The tree nodes are colour-coded by lineage, genotype, cgMLST HC200, HC100, and HC50 clusters (see legends). The scale bars indicate the number of cgMLST allelic differences. The interactive version of the tree is publicly available from https://enterobase.warwick.ac.uk/ms_tree?tree_id=92095

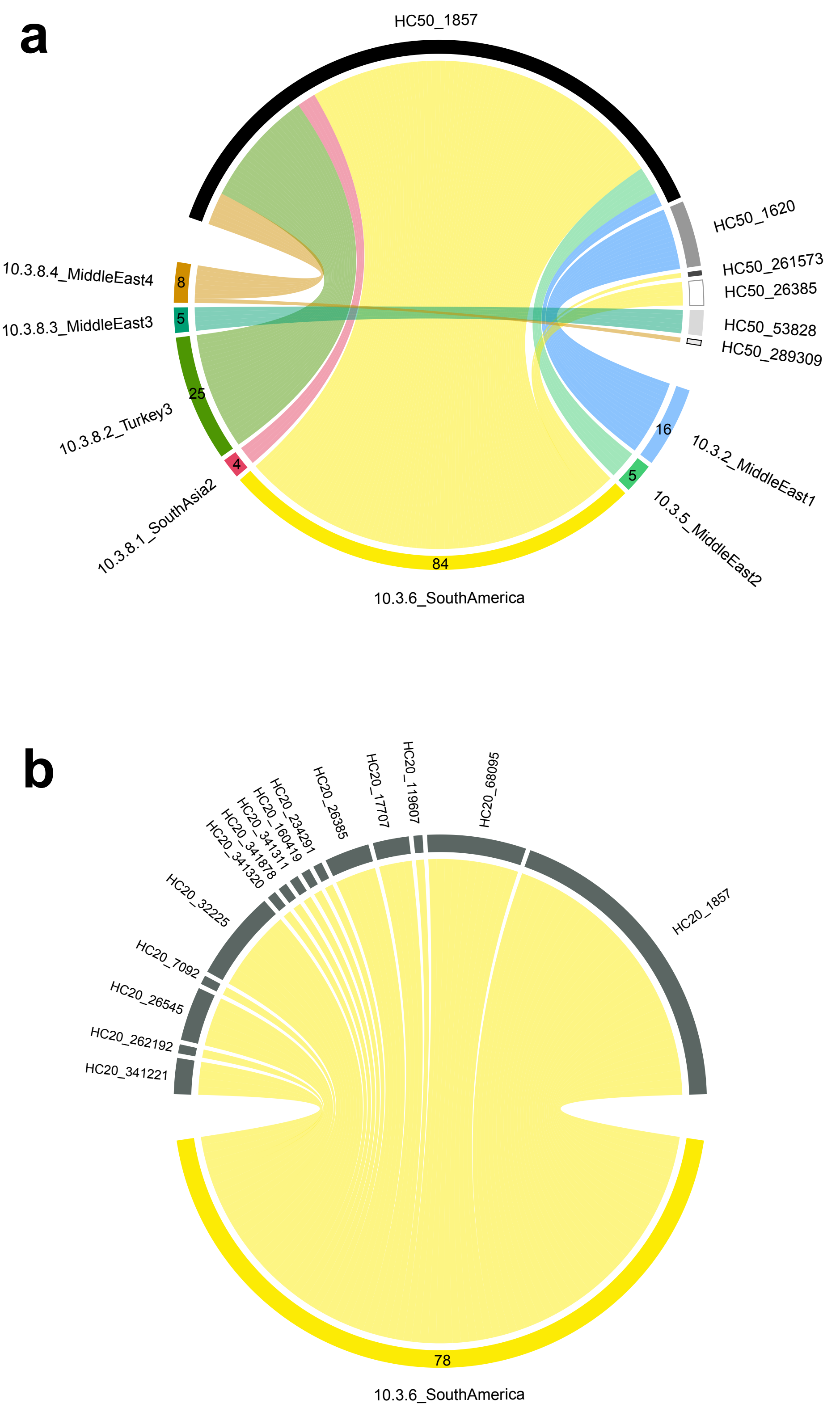

Supplementary Figure 7. Correlation between cgMLST and genotyping data for the tracking of particular strains. Circular plots illustrating the difficulty of using cgMLST HC50 (**a**) or HC20 (**b**) clustering to identify the most frequently isolated genotype, 10.3.6_SouthAmerica. The flow bars are coloured according to genotype. The number of isolates for each genotype is also indicated.

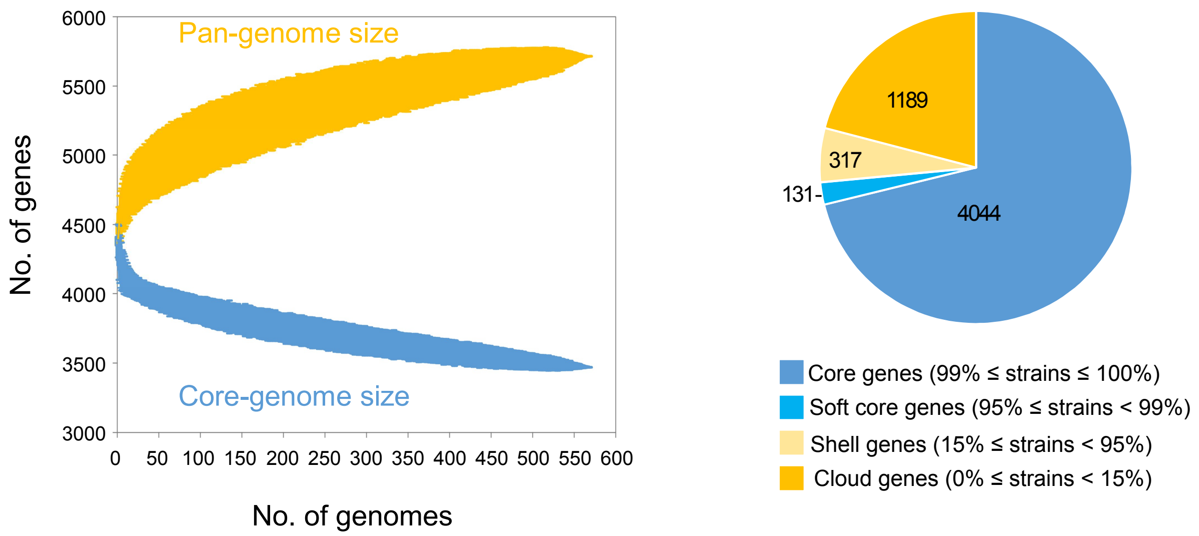

Supplementary Figure 8. Characteristics of the core- and pan-genomes of SPB$^-$ PG1. The pan-genome analysis was performed across the 11 SPB$^-$ PG1 lineages, with the assemblies of the 568 isolates of the diversity dataset. In the left panel, the pan-genome curve (dark yellow) shows the number of genes subsequently discovered as more genomes are added to the dataset. The rarefaction curve (blue) shows the decay in the number of core genes as more genomes are added to the dataset. Both pan-genome and core-genome curves were estimated from the panaroo binary matrix with PanGP[30] using a totally random sampling method and 10 sample repeats. The pie chart (right panel) shows the relative proportions of the core (dark blue), soft core (blue), shell (light yellow) and cloud (dark yellow) genes.

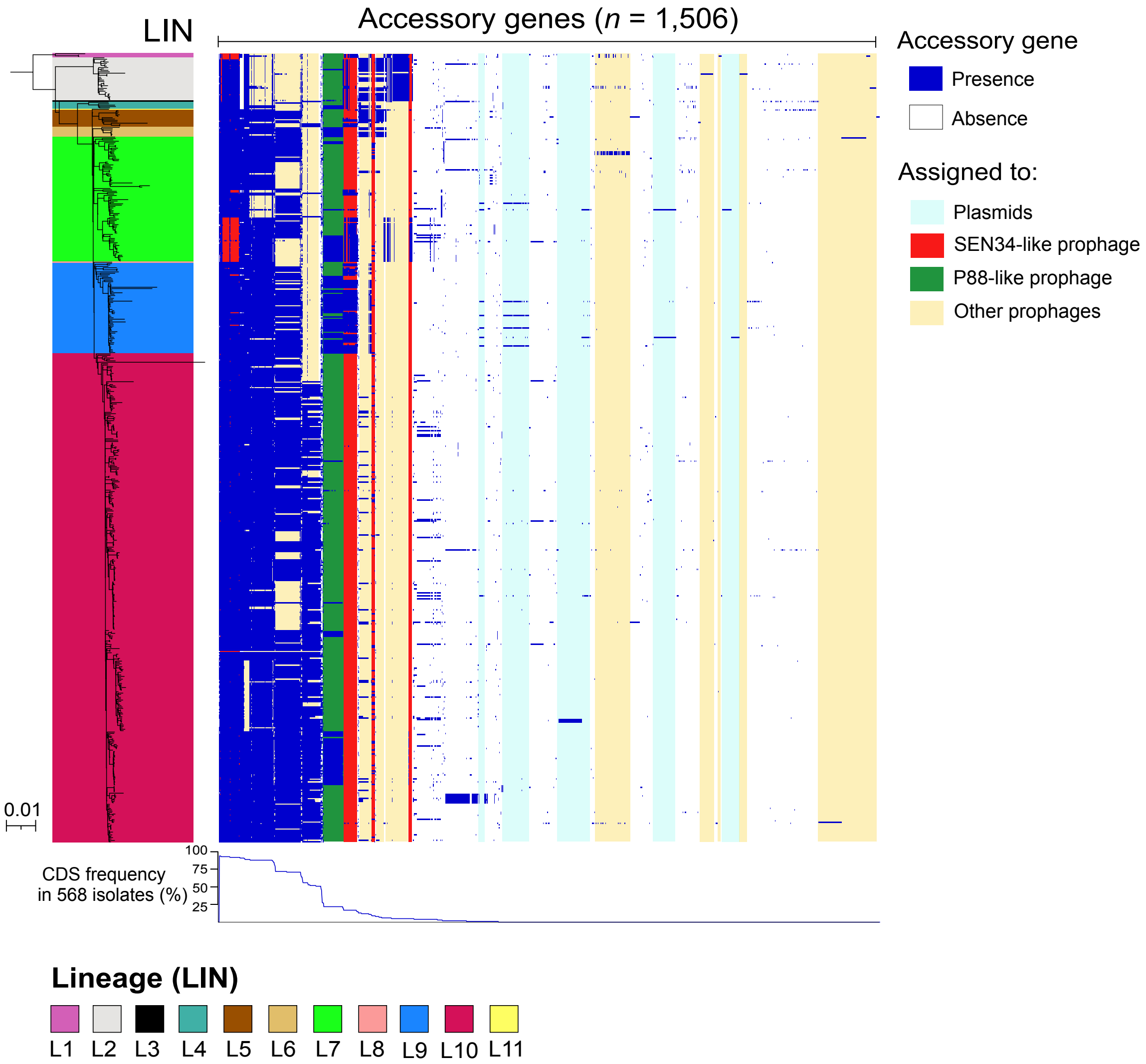

Supplementary Figure 9. Distribution of the 1,506 accessory genes across the phylogeny of SPB- PG1 and their attribution to prophages or plasmids. The pan-genome analysis was performed with the assemblies of the 568 isolates from the diversity dataset. The phylogeny is similar to that shown in Fig. 2a.

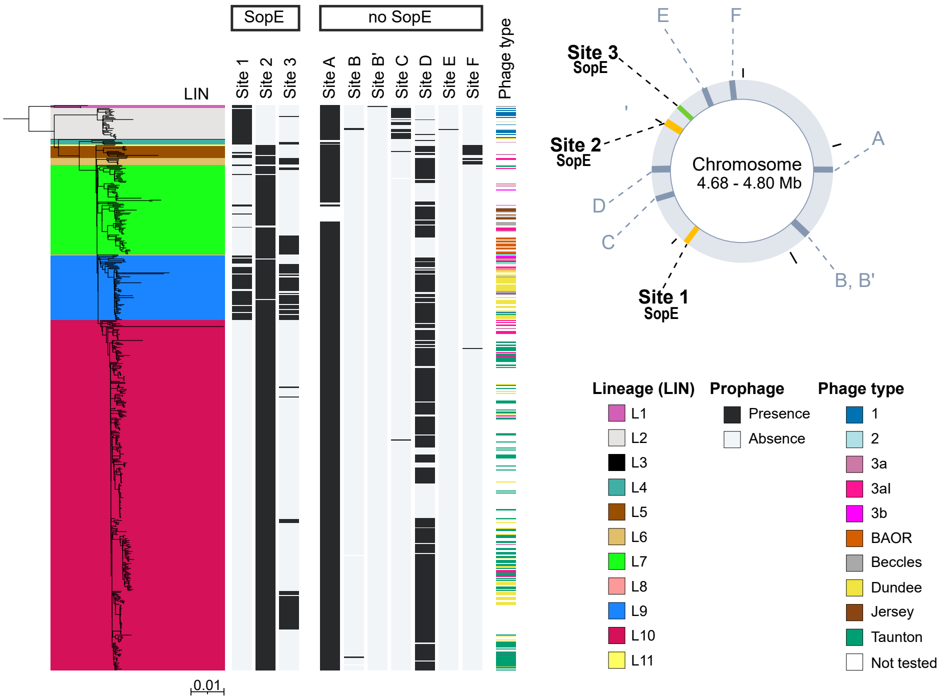

Supplementary Figure 10. Prophage content at 10 insertion sites (1-3, A, B, B', C-F) and phage types across the 11 lineages of SPB$^-$ PG1. Ten prophage insertion sites were identified from the comparative analysis of the 14 complete genomes (**Supplementary Data 8**). The occupancy of the 10 insertion sites was assessed across the 11 lineages of SPB$^-$ PG1, for the 568 isolates of the diversity dataset. Prophages at sites #1 to # 3 contain the *sopE* virulence gene. The schematic representation of the consensus chromosome starts at the *dnaA* locus (positions 1 to 1401).

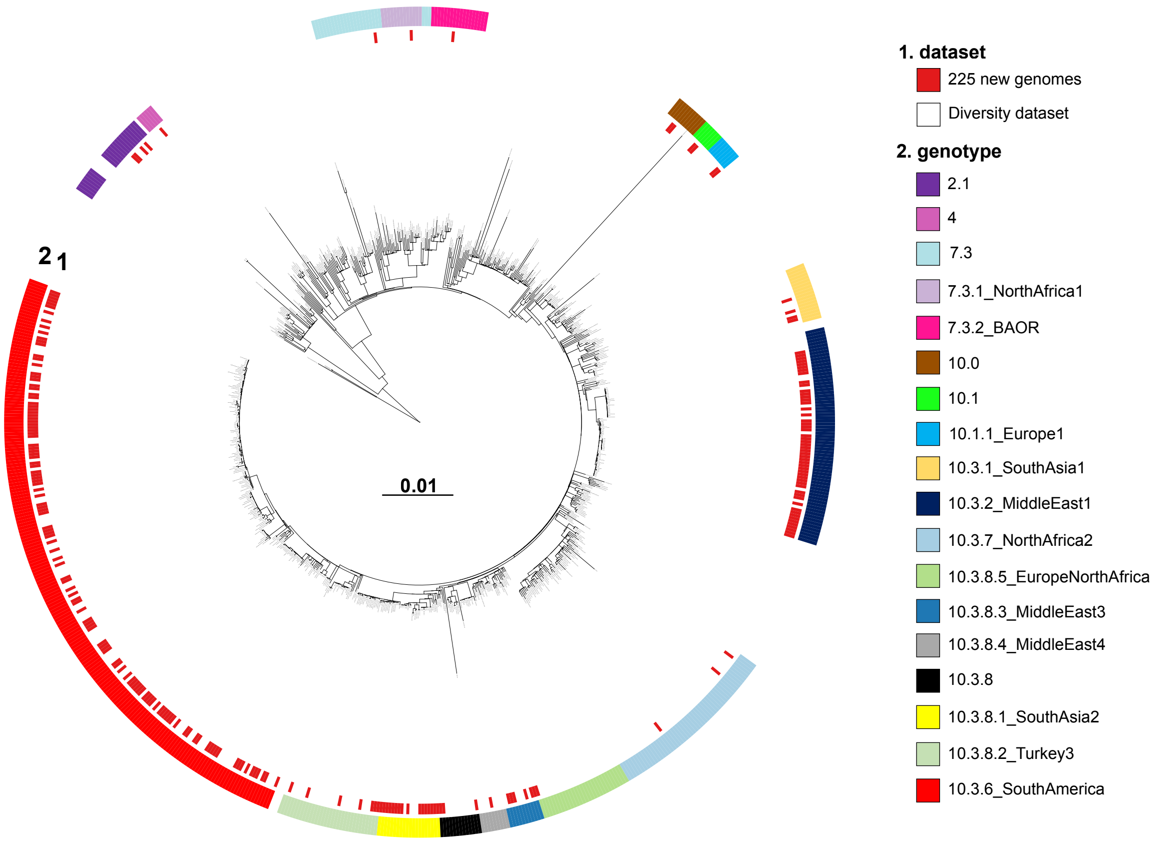

Supplementary Figure 11. Maximum likelihood phylogeny for 793 SPB$^-$ PG1 isolates. This phylogeny includes the 568 isolates of the diversity dataset (not coloured in ring 1) and 225 additional recent isolates (coloured in red in ring 1). For each isolate, its name, year of collection, country of origin, and source, are shown at the tips of the tree. The scale bar indicates the number of substitutions per variable site (SNV). The genotypes (see legend) of these additional 225 isolates are colour-coded in ring 2.

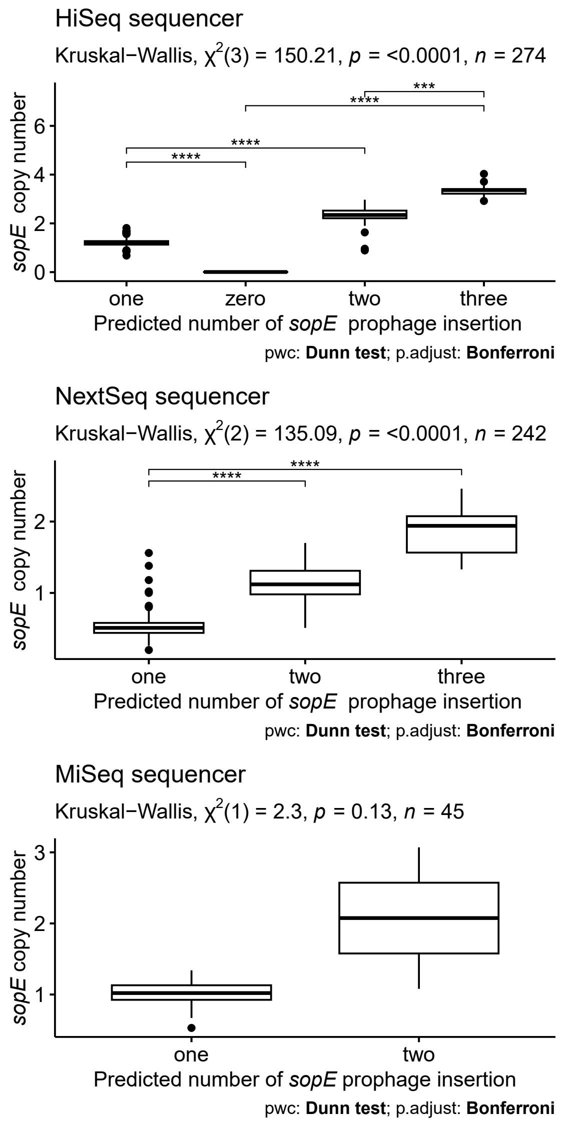

Supplementary Figure 12. Identification of the discrepancies between the predictions of *sopE* copy number and insertion site occupancy at sites #1, #2, and #3. Box plots visualise a summary of the data set (lower quartile, median, upper quartile) and dots correspond to outliers. The *sopE* copy number per genome was estimated from short-read mapping onto the B62 genome across the 11 SPB$^-$ PG1 lineages, for the 568 isolates of the diversity dataset. The distribution of *sopE* copy number values according to the number of *sopE* prophage insertion sites occupied can be used to detect potential discrepancies between site occupancy and *sopE* copy number predictions.

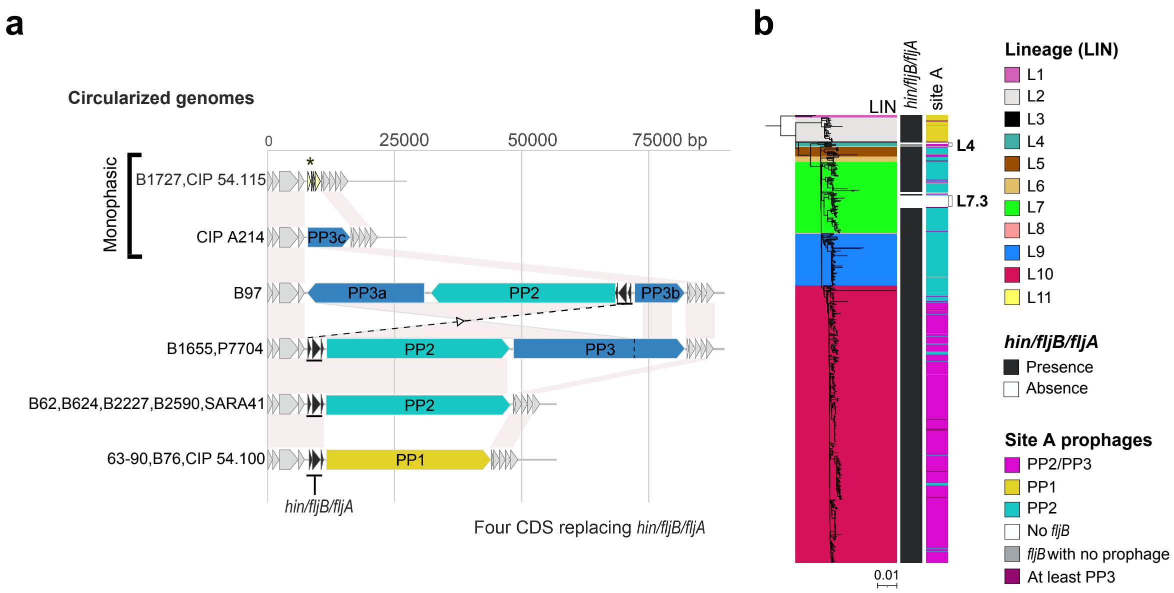

Supplementary Figure 13. Prophage insertion polymorphism in the close vicinity of the phase 2 flagellin gene (*fljB*) of SPB⁻ PG1. a, The *hin-fljB-fljA* gene cluster encoding the phase 2 flagellin was examined in detail in the 14 complete genomes. *hin* is the flagellar phase variation DNA invertase gene; *fljB* is the phase 2 flagellin gene; *fljA* is the phase 1 flagellin (*fliC*) repressor gene. The *hin-fljB-fljA* gene cluster is located between the *iroB* (encoding a salmochelin biosynthesis C-glycosyltransferase) and tmRNA-*ssrA* loci. The cluster is deleted in three isolates (B1727, CIP 54.115, CIP A214). The gene arrow maps illustrate the six genomic environments detected between *iroB* and tmRNA-*ssrA*. **b,** The presence of the *hin-fljB-fljA* gene cluster and prophage content near the *fljB* locus were determined across the 11 lineages of SPB$^-$ PG1, with the 568 isolates of the diversity set. Prophages were detected with short-read assemblies and the blastn algorithm. Further details are provided in **Supplementary Data 8.**

Supplementary References

1. Achtman, M., Hale, J., Murphy, R.A., Boyd, E.F. & Porwollik, S. Population structures in the SARA and SARB reference collections of *Salmonella enterica* according to MLST, MLEE and microarray hybridization. *Infect. Genet. Evol.* **16,** 314-325 (2013).
2. Pratt, J.H. On paratyphoid fever and its complications. *Boston Med. Surg.* **148,** 137-142 (1903).
3. Proescher, F. & Roddy, J.A. Bacteriological studies on paratyphoid A and paratyphoid B. *Arch. Intern. Med.* **3,** 263-312 (1910).
4. Boycott, A.E. Observations on the bacteriology of paratyphoid fever and on the reactions of typhoid and paratyphoid sera. *J. Hyg.* **6,** 33-73 (1906).
5. F. Rathery, F., Ambard, L., Vansteenberghe, P. & Michel, R. Les fièvres paratyphoïdes B à l'hôpital mixte de Zuydcoote, de décembre 1914 à février 1916. 1st ed. F. Alcan, Paris, 248pp (1916).
6. Gradmann, C., Harrison M., & Rasmussen, A. Typhoid and the military in the early 20th century. *Clin. Infect. Dis.* **69,** S385–S387 (2019).
7. Felix, A. & Callow, B.R. Typing of paratyphoid B bacilli by Vi bacteriophage. *Br. Med. J.* **2,** 127-130 (1943).
8. Savage, W. Paratyphoid fever: an epidemiological study. *J. Hyg.* **42,** 393-410 (1942).
9. Sharp, J.C., Brown, P.P. & Sangster, G. Outbreak of paratyphoid in Edinburgh area. *Br. Med. J.* **1,** 1282-1285 (1964).
10. Newell, K.W., Hobbs, B.C. & Wallace, E.J. Paratyphoid fever associated with Chinese frozen whole egg; outbreaks in two bakeries. *Br. Med. J.* **2,** 1296-1298 (1955).
11. Sloan, R.S., Wilson, H.D. & Wright, H.A. The detection of a carrier of multiple phage-types of *Salmonella paratyphi B*. *J. Hyg.* **58,** 193-200 (1960).
12. Connor, T.R. *et al*. What's in a name? Species-wide whole-genome sequencing resolves invasive and noninvasive lineages of *Salmonella enterica* serotype Paratyphi B. *mBio* **7,** e00527-16 (2016).
13. Alikhan, N.F., Zhou, Z., Sergeant, M.J. & Achtman, M. A genomic overview of the population structure of *Salmonella*. *PLoS Genet.* **14,** e1007261 (2018).
14. Zhou, Z., Alikhan, N.F., Mohamed, K., Fan, Y.; Agama Study Group & Achtman, M. The EnteroBase user's guide, with case studies on *Salmonella* transmissions, *Yersinia pestis* phylogeny, and *Escherichia* core genomic diversity. *Genome Res.* **30,** 138-152 (2020).
15. Zhou, Z., Charlesworth, J. & Achtman, M. HierCC: a multi-level clustering scheme for population assignments based on core genome MLST. *Bioinformatics* **37,** 3645-3646 (2021).
16. Malorny, B., Bunge, C. & Helmuth, R. Discrimination of d-tartrate-fermenting and -nonfermenting *Salmonella enterica* subsp. *enterica* isolates by genotypic and phenotypic methods. *J. Clin. Microbiol.* **41,** 4292-4297 (2003).
17. Achtman, M., Hale, J., Murphy, R.A., Boyd, E.F. & Porwollik, S. Population structures in the SARA and SARB reference collections of *Salmonella enterica* according to MLST, MLEE and microarray hybridization. *Infect. Genet. Evol.* **16,** 314-325 (2013).
18. Kelterborn, E. *Salmonella*-species. Erstfunde, Namen und Vorkommen. Den Haag (Junk) 535 pp (1967).
19. Aoki, Y. Distribution of *Salmonella* Types in East Asia. *Endemic Diseases Bulletin of Nagasaki University* **7,** 192-220 (1965).

20. Achtman M. *et al*. Multilocus sequence typing as a replacement for serotyping in *Salmonella enterica*. *PLoS Pathog.* **8,** e1002776 (2012).
21. Longfellow, D. & Luippold, G.F. Typhoid Vaccine Studies VII: Typhoid-Paratyphoid Vaccine. *Am. J. Public Health Nations Health* **33,** 561-568 (1943).
22. Gard, S. Ein neuer Salmonella-Typ (S. abortus canis). *Zeitschr. f. Hygiene*. **121,** 139-141 (1938).
23. Magnusson, K.E. Ein Hund als Ansteckungsquelle von Paratyphusinfektionen. Zeitschr. f. Hygiene. **121,** 136–138 (1938).
24. Allos, G. Inventaire des sérotypes de *Salmonella* rencontrés en Irak. *Bull. Soc. Pathol. Exot. Filiales* **71,** 323-328 (1978).
25. Felix A. World survey of typhoid and paratyphoid-B phages types. *Bull. World Health Organ.* **13,** 109-170 (1955).
26. Nicolle P. Rapport sur la distribution des lysotypes de *Salmonella typhi* et de *S. paratyphi B* dans le monde, d'après les résultats fournis par les centres nationaux membres du comité international de la lysotypie entérique à l'occasion du congrès international de microbiologie, Stockholm, 1958. *Ann. Inst. Pasteur* **102,** 389-409 (1962).
27. International Committee for Enteric Phage-Typing (ICEPT). The geographical distribution of *Salmonella typhi* and *Salmonella paratyphi A* and *B* phage types during the period 1 January 1966 to 31 December 1969. *J. Hyg.* **71,** 59-84 (1973).
28. Vieu, J.F., Binette, H. & Leherissey, M. Salmonella paratyphi B d-tartrate positif (var. java): lysotypie de 1200 souches isolées en France (1975-1985). *Zentralbl. Bakteriol. Mikrobiol. Hyg. A*. **268,** 424-432 (1988).
29. Fukumi, H. *et al.* Epidemiological investigations of typhoid and paratyphoid fever in Japan with aid of the phage typing method. I. Distribution of phage-types of *Salmonella typhi* and *Salmonella paratyphi B* in Japan, 1956-1965. *Jpn J. Med. Sci. Biol.* **20,** 447-460 (1967).
30. Zhao, Y. *et al.* PanGP: A tool for quickly analyzing bacterial pan-genome profile. *Bioinformatics* **30**, 1297–1299 (2014).
