# Supplementary material for: Genomic perspective on the bacillus causing paratyphoid B fever
Source: Nat Commun. 2024 Dec 10;15:10143. doi: 10.1038/s41467-024-54418-4 (PMC11632088; doi:10.1038/s41467-024-54418-4)
Supplement: Supplementary file 3 — Description of Additional Supplementary Files [file 41467_2024_54418_MOESM3_ESM.pdf]

Description of Additional Supplementary Files

Supplementary Data 1
Description: Metadata for the 568 SPB$^-$ PG1 isolates and genomes of the diversity dataset and the two outgroup isolates.

Supplementary Data 2
Description: Details for the 15,995 single nucleotide variants (SNVs) used in the maximum likelihood phylogeny of the 568 SPB$^-$ PG1 genomes.

Supplementary Data 3
Description: The binary pan-matrix generated for the 568 SPB$^-$ PG1 isolates of the diversity dataset. The gene_presence_absence matrix generated using the Panaroo software, is in the format given by Roary (Page, A. J. *et al.* Roary: rapid large-scale prokaryote pan genome analysis. *Bioinformatics* 31, 3691–3693 (2015)), with the presence and absence of genes in each sample being binary coded, 1 for presence.

Supplementary Data 4
Description: Assignment of the 1,506 accessory genes to plasmids, prophages and transposases.

Supplementary Data 5
Description: Details for the 38 marker single nucleotide variants (SNVs) used for the SPB$^-$ PG1 genotyping scheme.

Supplementary Data 6
Description: Metadata for the 336 SPB$^-$ PG1 isolates and genomes of the surveillance dataset.

Supplementary Data 7
Description: Correction of some data (MLST, phylogroup, serotype) for SPB$^-$ and SPB$^+$ isolates published in the Table S1 of the study by Connor *et al.* mBio. 2016.

Supplementary Data 8
Description: Coordinates of insertion sites and characteristics of the prophages identified in the 14 SPB$^-$ PG1 complete genomes.

Supplementary Data 9
Description: The *sopE* gene copy number and prophage insertion sites' occupancy in the 568 SPB$^-$ PG1 genomes of the diversity dataset.

Supplementary Data 10
Description: List of the 102 non-SPB$^-$ PG1 *Salmonella enterica* reference genomes used to assess the genotyping tool for SPB$^-$ PG1.
