# Supplementary material for: Genomic perspective on the bacillus causing paratyphoid B fever
Source: Nat Commun. 2024 Dec 10;15:10143. doi: 10.1038/s41467-024-54418-4 (PMC11632088; doi:10.1038/s41467-024-54418-4)
Supplement: Supplementary file 14 — Reporting Summary [file 41467_2024_54418_MOESM14_ESM.pdf]

Corresponding author(s): FX Weill

Last updated by author(s): Oct 30, 2024

Reporting Summary

Nature Portfolio wishes to improve the reproducibility of the work that we publish. This form provides structure for consistency and transparency in reporting. For further information on Nature Portfolio policies, see our Editorial Policies and the Editorial Policy Checklist.

Statistics

For all statistical analyses, confirm that the following items are present in the figure legend, table legend, main text, or Methods section.

n/a	Confirmed	
☐	☒	The exact sample size (*n*) for each experimental group/condition, given as a discrete number and unit of measurement
☒	☐	A statement on whether measurements were taken from distinct samples or whether the same sample was measured repeatedly
☐	☒	The statistical test(s) used AND whether they are one- or two-sided<br>*Only common tests should be described solely by name; describe more complex techniques in the Methods section.*
☒	☐	A description of all covariates tested
☐	☒	A description of any assumptions or corrections, such as tests of normality and adjustment for multiple comparisons
☐	☒	A full description of the statistical parameters including central tendency (e.g. means) or other basic estimates (e.g. regression coefficient) AND variation (e.g. standard deviation) or associated estimates of uncertainty (e.g. confidence intervals)
☒	☐	For null hypothesis testing, the test statistic (e.g. *F*, *t*, *r*) with confidence intervals, effect sizes, degrees of freedom and *P* value noted<br>*Give P values as exact values whenever suitable.*
☐	☒	For Bayesian analysis, information on the choice of priors and Markov chain Monte Carlo settings
☒	☐	For hierarchical and complex designs, identification of the appropriate level for tests and full reporting of outcomes
☒	☐	Estimates of effect sizes (e.g. Cohen's *d*, Pearson's *r*), indicating how they were calculated

Our web collection on statistics for biologists contains articles on many of the points above.

Software and code

Policy information about availability of computer code

Data collection	Excel version 16; EnteroBase (https://enterobase.warwick.ac.uk/species/index/senterica), CARD v.3.0.8 AMR gene database (https://card.mcmaster.ca/)
Data analysis	Kraken v.2.1.1 (https://ccb.jhu.edu/software/kraken/), HGAP workflow (https://rhallpb.github.io/Applications/HGAP.html), Pilon v.1.23 (https://github.com/broadinstitute/pilon), Guppy v.4.3.4 or v.5.0.13 (https://pypi.org/project/ont-pyguppy-client-lib/), filtlong tool v.0.2.1 (https://github.com/rrwick/Filtlong/), Flye v.2.9 (https://github.com/fenderglass/Flye), raven v.1.6.0 (https://github.com/lbcb-sci/raven), miniasm v.0.3 (https://github.com/lh3/miniasm), Minipolish v.0.1.3 (https://github.com/rrwick/Minipolish), medaka v.1.4.4 (https://github.com/nanoporetech/medaka), bakta v.1.5.0 (https://github.com/oschwengers/bakta), KMA v.1.4.14 (https://tracker.debian.org/pkg/kma), BLAST+ v.2.14.1 (https://ftp.ncbi.nlm.nih.gov/blast/executables/blast+/2.14.1/), GrapeTree (https://github.com/achtman-lab/GrapeTree), SRST2 v.0.2.0 (https://github.com/katholt/srst2), PlasmidFinder v.2.0 (https://cge.food.dtu.dk/services/PlasmidFinder-2.0/), RedDog (https://github.com/katholt/reddog-nf), Gubbins v.2.3.2 (https://github.com/nickjcroucher/gubbins/tree/master), RAxML v.8.2.9 (https://github.com/stamatak/standard-RAxML), iTOL v.6 (https://itol.embl.de), TempEst v.1.5.3 (http://tree.bio.ed.ac.uk/software/tempest/), BEAST v.2.7.1 (https://github.com/CompEvol/beast2/releases), dendropy v.4.5.2 (https://pypi.org/project/DendroPy/), Tracer v.1.7.2 (https://github.com/beast-dev/tracer/releases/tag/v1.7.2), fastbaps v.1.0.8 (https://github.com/gtonkinhill/fastbaps), SNPPar v.1.1 (https://github.com/d-j-e/SNPPar), IQTree v.2 (http://www.iqtree.org/), fq2dna/21.06 script (https://gitlab.pasteur.fr/GIPhy/fq2dna), SPAdes v.3.15.5 (https://github.com/ablab/spades), panaroo v.1.3.0 (https://github.com/gtonkinhill/panaroo), micropan v.2.1 (https://www.rdocumentation.org/packages/micropan/versions/2.1), PHASTER (https://phaster.ca/), bowtie2 v.2.3.5.1 (https://github.com/BenLangmead/bowtie2/releases), samtools v.1.13 (https://github.com/samtools/samtools/releases/), Tablet v.1.21.02.08 (https://ics.hutton.ac.uk/tablet/), blastn (https://blast.ncbi.nlm.nih.gov/Blast.cgi), gggenes v.0.5.0 (https://github.com/wilkox/gggenes), ggplot2 v.3.4.2 (https://github.com/tidyverse/ggplot2), R v.4.1.2 (https://www.npackd.org/p/r/4.1.2; https://cran.r-project.org/doc/manuals/), rstatix v.0.7.2 (https://github.com/kassambara/rstatix), ggpubr v.0.6.0 (https://github.com/kassambara/ggpubr), Mykrobe v.0.12.2 (https://

github.com//mykrobe-tools/mykrobe) with the Paratyphi B panel (https://figshare.com/articles/dataset/Mykrobe_panel_paratyphi_B_version_20230627/24925506).

For manuscripts utilizing custom algorithms or software that are central to the research but not yet described in published literature, software must be made available to editors and reviewers. We strongly encourage code deposition in a community repository (e.g. GitHub). See the Nature Portfolio guidelines for submitting code & software for further information.

Data

Policy information about availability of data

All manuscripts must include a data availability statement. This statement should provide the following information, where applicable:

- Accession codes, unique identifiers, or web links for publicly available datasets
- A description of any restrictions on data availability
- For clinical datasets or third party data, please ensure that the statement adheres to our policy

The publicly available sequences used in this study are available from GenBank (https://www.ncbi.nlm.nih.gov/genbank/) under accession numbers NC_028699, NC_026014, NC_010102.1, CP074225.1, L78932, NZ_CP065185, NZ_CP065186.1, NZ_CP065187.1, NZ_CP065188.1, JWQX00000000.1.

The long-read sequence data generated in this study are available from GenBank under accession numbers CP147895, CP147896, CP147897, CP147898, CP147899, CP147900, CP147901, CP147902, CP147903, CP147904, CP147905, CP147906, and CP147907. The short-read sequence data generated in this study were submitted to EnteroBase (https://enterobase.warwick.ac.uk/) and to the European Nucleotide Archive (ENA, https://www.ebi.ac.uk/ena/) under study numbers PRJDB11608, PRJEB18998, PRJEB28356, PRJEB30317, PRJEB67705, PRJNA248792, PRJEB68323, PRJEB49424, PRJEB71958. All the accession numbers of the short-read sequences produced and/or used in this study are listed in Supplementary Data 1 and Supplementary Data 6.

The list of genomes studied (and their assembled short-read data) can be obtained from EnteroBase at: https://enterobase.warwick.ac.uk/species/senterica/search_strains?query=workspace:86468 (diversity dataset, Supplementary Data 1) and: https://enterobase.warwick.ac.uk/species/senterica/search_strains?query=workspace:91222 (surveillance data set, Supplementary Data 4).

The list of 166 genomes (and their assembled short-read data) published by Connor et al.27 and present in EnteroBase can be obtained from https://enterobase.warwick.ac.uk/species/senterica/search_strains?query=workspace:86472.
The cgMLST GrapeTrees shown in Supplementary Figs. 1 and 7 can be visualised with EnteroBase at: https://enterobase.warwick.ac.uk/ms_tree?tree_id=92077 and https://enterobase.warwick.ac.uk/ms_tree?tree_id=92095, respectively.

Source data are provided with this paper.

Research involving human participants, their data, or biological material

Policy information about studies with human participants or human data. See also policy information about sex, gender (identity/presentation), and sexual orientation and race, ethnicity and racism.

Reporting on sex and gender	Not relevant
Reporting on race, ethnicity, or other socially relevant groupings	Not relevant
Population characteristics	This study was based exclusively on bacterial isolates (including historical and reference strains) and associated metadata. The vast majority of these isolates (n = 362) were obtained from Institut Pasteur (reference laboratories or Collection de l'Institut Pasteur (CIP)); 115 isolates corresponded to historical or reference strains collected between 1898 and 1971 and 247 were bacterial isolates collected under the mandate for laboratory-based surveillance awarded by the French Ministry of Health to the National Reference Centre for Escherichia coli, Shigella and Salmonella (NRC-ESS) since 1972. Data collection and storage by the NRC-ESS was approved by the French National Commission for Data Protection and Liberties ("Commission Nationale Informatique et Libertés (CNIL)"; approval number: 1474659). For other human bacterial isolates collected (or the genome sequences derived from them) by participating reference laboratories under local mandates for laboratory-based surveillance of salmonellosis in line with local laws and regulations, the associated metadata did not contain any personal identifiable information and were restricted to year and country of isolation, and international travel information.
Recruitment	No recruitment of patients
Ethics oversight	As this study was based exclusively on bacterial strains collected by national reference centers under local mandates for laboratory-based surveillance of salmonellosis, as a result neither informed consent nor approval from an ethics committee was sought given this is not research conducted on human participants.

Note that full information on the approval of the study protocol must also be provided in the manuscript.

Field-specific reporting

Please select the one below that is the best fit for your research. If you are not sure, read the appropriate sections before making your selection.

☒ Life sciences ☐ Behavioural & social sciences ☐ Ecological, evolutionary & environmental sciences

Life sciences study design

All studies must disclose on these points even when the disclosure is negative.

Sample size	We have analysed the genomic sequences of 568 Salmonella enterica Paratyphi B strains isolated betwen 1898 and 2021 in 41 countries to cover the broadest diversity in terms of sources and temporo-spatial distribution: 446 of these strains (originating from different reference laboratories across the world) were sequenced specifically for this study and 109 had already been published. Limitations were imposed by available resources and funding, and also by the fact that not all of the archived cultures were viable. No statistical methods were used to predetermine sample size, which eventually was larger than in similar studies dealing with particular populations of enteric bacterial pathogens (Zhou et al PNAS 2014, Njamkepo et al. Nat Microbiol 2016, Cuypers et al. Nat Commun 2024).
Data exclusions	Twelve genomic sequences were removed due to the following reasons: genomic sequences that did not pass quality control (n = 7), duplicate isolates (n = 2), isolates not belonging to PG1 (n = 2), and one isolate recovered from a culture of uncertain origin (n = 1).
Replication	The maximum likelihood phylogenetic tree was successfully replicated four times.
Randomization	Our 568 genomes were not allocated into different experimental groups (observational study). Therefore, randomization was not used in this study.
Blinding	For the phylogenetic analysises and genomic analysis of antibiotic resistance determinants, only the isolate name was used as identifier. Therefore we were "blind" to the associated metadata before comparative analysis.

Reporting for specific materials, systems and methods

We require information from authors about some types of materials, experimental systems and methods used in many studies. Here, indicate whether each material, system or method listed is relevant to your study. If you are not sure if a list item applies to your research, read the appropriate section before selecting a response.

Materials & experimental systems

n/a	Involved in the study	
☒	☐	Antibodies
☒	☐	Eukaryotic cell lines
☒	☐	Palaeontology and archaeology
☒	☐	Animals and other organisms
☒	☐	Clinical data
☒	☐	Dual use research of concern
☒	☐	Plants

Methods

n/a	Involved in the study	
☒	☐	ChIP-seq
☒	☐	Flow cytometry
☒	☐	MRI-based neuroimaging

Plants

Seed stocks	*Report on the source of all seed stocks or other plant material used. If applicable, state the seed stock centre and catalogue number. If plant specimens were collected from the field, describe the collection location, date and sampling procedures.*
Novel plant genotypes	*Describe the methods by which all novel plant genotypes were produced. This includes those generated by transgenic approaches, gene editing, chemical/radiation-based mutagenesis and hybridization. For transgenic lines, describe the transformation method, the number of independent lines analyzed and the generation upon which experiments were performed. For gene-edited lines, describe the editor used, the endogenous sequence targeted for editing, the targeting guide RNA sequence (if applicable) and how the editor was applied.*
Authentication	*Describe any authentication procedures for each seed stock used or novel genotype generated. Describe any experiments used to assess the effect of a mutation and, where applicable, how potential secondary effects (e.g. second site T-DNA insertions, mosiacism, off-target gene editing) were examined.*
